# Women’s information needs about menopause: a cross-sectional survey in Norwegian general practice

**DOI:** 10.3399/BJGPO.2023.0127

**Published:** 2024-01-24

**Authors:** Marianne Natvik, Bjorn Gjelsvik, Siri Vangen, Holgeir Skjeie, Mette Brekke

**Affiliations:** 1 Department of General Practice, Faculty of Medicine, University of Oslo, Oslo, Norway; 2 Norwegian Research Centre for Women’s Health, Oslo University Hospital & Clinical Medicine, University of Oslo, Norway; 3 General Practice Research Unit, University of Oslo, Norway

**Keywords:** menopause, women’s health, health education, general practice

## Abstract

**Background:**

Research has indicated that providing women with information about menopause can improve their attitudes towards it and symptom experience. Nevertheless, information shared on the menopause is often arbitrary.

**Aim:**

To examine women’s information needs about menopause, and understand if, when, and from whom they want information.

**Design & setting:**

A cross-sectional study was undertaken. A questionnaire survey was distributed to women in the waiting room of 54 general practice clinics in South-Eastern Norway in autumn 2022.

**Method:**

Medical students recruited women in the clinic waiting rooms. A 1-page study-specific questionnaire was used, focusing on need for information about menopause. A multinominal logistic regression model was used to analyse the association between the desire for information and education level, country of birth, and menopausal status.

**Results:**

A total of 625 women were included, with a mean age of 44.4 years (standard deviation [SD] 8.7). In all, 59% answered that they wanted information about menopause, and 81% of these wanted their GP to inform them, from a median age of 45 years. According to the women, only 10% of GPs had initiated a discussion on the menopause. Higher education was a predictor for wanting information. A main driver of information needs was to help oneself in the present and in the future. In all, 33% did not want information. The main reasons were that they already possessed sufficient information, would take menopause as it comes, were too young, or were already postmenopausal. The sex of the GP did not influence the results.

**Conclusion:**

Most women wanted information about menopause from their GP. The study emphasises the need for GPs to consider prioritising this discussion, and to keep up to date on recommendations and treatment options.

## How this fits in

Menopause is not a disease; however, women may experience symptoms that can leave them feeling uncertain or unwell. Adequate knowledge about menopause can aid women in recognising the symptoms, which can help to equip them to manage it. Previous studies have shown that women want information about the menopause; this research has provided support for these findings. It has shown that about two-thirds of women want such information and consider their GP important in providing it. The participants wanted information before reaching menopause, from aged 45 years.

## Introduction

Women experience natural menopause between the ages of 45 and 55 years.^1^ In high-income countries, they thus live one-third of their life after menopause. Although every woman goes through menopause, it is often a theme that women, doctors, and society tends to avoid discussing. Menopause is not a disease, but the symptoms a woman experiences might make her feel ill, and affect her function and quality of life.^2,3^ A woman’s experience of menopause is influenced by her symptoms, views, knowledge about this period, and the support she receives if she seeks help.^4,5^ Lack of knowledge and misleading assumptions about menopause can lead to a sense of uncertainty regarding the experiences a woman goes through.^4–6^ Prior information about menopause can support women’s attitudes towards it and their symptom experience.^4,7^ Yet there is no system for distributing such information to all women.^5,8^ Information about menopause, and the changes in the postmenopausal body, is important to optimise health throughout the rest of a woman’s life.^8–10^

Society’s focus on menopause is increasing and knowledge is becoming more available, but it is of varying quality and is, at times, confusing to women and even healthcare professionals.^5,10–14^ As a result, more women may consult their GP for up-to-date information about this topic.^11^ This requires time in the general practice, and that the GP is updated on the menopausal treatment and advice.^8,15^ GPs’ attitudes towards menopause as a natural phase in every woman’s life, and their views on the risk associated with menopausal hormone treatment (MHT; hormone replacement therapy [HRT] in the UK), may make GPs reluctant to prescribe MHT.^15,16^ The view that menopause is a ‘long-term female hormone deficiency’ may lead women to believe that MHT is a ‘wonder drug’.^11^ These different views may result in conflicting expectations. Time-constricted GPs may feel they spend time talking to healthy women, with insufficient time to talk to other patients,^11^ and they express that scarcity of time limits a holistic approach to managing menopause.^15^

Female patients in Norway visit their GP on average 3.6 times yearly.^17^ The GP is the first contact point for most medical issues and is a gatekeeper to gynaecologists.^18,19^ For reasons of health economy and resources, GPs should meet women’s needs concerning menopause appropriately.^20^

Knowledge about how, when, and why women want information about menopause is limited. We need to learn how women in Norway acquire this information and whether they find it helpful, and their expectations regarding the role of GPs in providing information. This study aims to investigate these issues.

## Method

### Study design, setting, participants, and data collection

A cross-sectional questionnaire survey was conducted during autumn in 2022. It was undertaken in the waiting rooms of 54 GP practices in South-Eastern Norway. During deployment in general practice, medical students at the University of Oslo distributed and collected the questionnaires. Fifty-four students participated in our study. The students were offered a reward for the task. The women were not offered compensation for participating in the study.

The questionnaire was a 1-page form on paper. The women answered while waiting for their GP. The students collected the questionnaires and sent or delivered them in a sealed envelope to the Department of General Practice at the University of Oslo. The form was filled out anonymously. In total, 959 women were asked to participate, and 86% (*n* = 829) accepted. The response rate was calculated from reports made by 44 of the 54 students, who systematically registered the women they asked to participate. The inclusion criteria were women aged between 30–60 years who could read Norwegian. Questionnaires from older or younger responders (*n* = 184) and questionnaires without the woman’s menopausal status (*n* = 3) were excluded. Questionnaires completed inconsistently (*n* = 9) or revealing that the woman had breast cancer or gynaecological diseases (*n* = 8) were also excluded. In total, 204 questionnaires were excluded. A total of 625 women were included in the analysis.

### Contents of the questionnaire

The research group created a study-specific questionnaire, as we could not find a suitable validated alternative. The questionnaire used by Cooper in their research on factors that influence women’s search for information about menopause inspired us.^6^ The questionnaire was validated through discussions with 18 women in a GP practice to confirm the wording, avoid misunderstandings, and secure the questionnaire flow. It was also discussed with GP colleagues and researchers.

The questionnaire contained three parts, with 23 questions. (See Supplementary data files.)

The first part included background information. The second part was about women’s need for information and if they had searched for information themselves. The third part concerned women’s experiences and preferences for involving the GP in giving information. Most questions included multiple-answer options, some with the opportunity to provide free-text responses. One question was exclusively free text (‘*If you have searched for information about menopause, from where did you get it*?’).

### Statistical analysis

The women’s demographics and the results are presented with mean and standard deviation (SD) for normally distributed data, or otherwise median. For categorical variables, frequencies and percentages were calculated. A multinominal logistic regression model was used to analyse the association between the desire for information and education level, country of birth, and menopausal status. The association was presented as a relative risk ratio (RRR) with 95% a confidence interval (CI). Two steps preceded the modelling. First, unadjusted models were fitted to the data. Second, all independent variables with *P*<0.2 from the unadjusted models were used to fit the adjusted model.

The questionnaires were registered in an online form and transferred to SPSS. The statistical analysis was done using SPSS Statistics (version 29).

## Results

### Demographics

The characteristics of the responders are summarised in Table 1. Among the 625 women included, 58% defined themselves as premenopausal, 24% were in the menopausal transition, 8% were postmenopausal, and 9% did not know. The participants were born in 43 different countries, the majority in Norway. The country of origin of their mothers showed the same distribution. Sixty-one per cent had higher education (>12 years). Of the participants’ GPs, 46% were female and 53% male (1% did not know their GP’s sex).

**Table 1.** Characteristics of responders (*n* = 625)

Age	Mean, SD
Age, years	44.4 (8.66)
**Country or continent of origin**	***n* (%**)
Norwegian	523 (83.9)
Western Europe and USA	31 (5.0)
Eastern Europe	30 (4.8)
Asia, Africa, or South America	39 (6.3)
Missing	2
**Education**	
Lower education ≤12 years	242 (39.0)
Higher education >12 years	379 (61.0)
Missing	4
**Menopausal status**	
Premenopausal	363 (58.1)
In menopausal transition	152 (24.3)
Postmenopausal	52 (8.3)
Do not know if in menopause	58 (9.3)

### Information needs and preferences

We found that almost two-thirds of the participants (59%) wanted information about menopause (Table 2 and Supplementary Table S1). Among all women, the main reason for information need was ‘*I want to know how to help myself*’ in this period, as stated by 62% (Table 3). The main reason for information for participants who were premenopausal was *‘I want to prepare myself for the menopausal transition’* (70%), and 40% said they were curious about menopause. Participants in the menopausal transition wanted information because they had symptoms (61%) or wanted to know more about symptoms (45%) or treatment (55%), and 79% wanted information to know how to help themselves.

**Table 2.** Information need about menopause according to menopausal status, education level, and country of origin. Number, per cent, and relative risk ratio (RRR) are presented

	Yes		No		Unsure			
Information need	*n*	%	*n*	%	*n*	%	RRR,^a,b^95% CI	^2^RRR, ^b,c^95% CI
All	366	58.6	205	32.9	52	8.3	1.79(1.50 to 2.12)	
**Menopausal status**								
Premenopausal	217	59.9	106	29.3	39	10.8	Reference	Reference
In menopausal transition	94	61.8	49	32.2	9	5.9	0.94(0.62 to 1.42)	1.04(0.68 to 1.59)
Postmenopausal	12	23.1	38	73.1	2	3.8	0.15(0.08 to 0.31)^d^	0.16(0.08 to 0.33)^d^
Do not know if in menopause	43	75.4	12	21.1	2	3.5	1.75(0.89 to 3.46)	1.85(0.93 to 3.68)
**Education**								
Lower education	121	50.0	91	37.6	30	12.4	0.62(0.44 to 0.88)^e^	0.62(0.43 to 0.90)^e^
Higher education^f^	242	64.2	113	30	22	5.8	Reference	

^a^Unadjusted model. ^b^Information need: yes versus no (no as the referent). ^c^Adjusted model based on independent variables purposeful selected (*P*<0.2) from the unadjusted model. ^d^ *P*<0.001. ^e^ *P*<0.05. ^f^Missing *n* = 2.

**Table 3.** Reasons for wanting or not wanting information about menopause among all women

Why do you want information? (*n* = 366)	%
I want to know how to help myself	62.0
I want to prepare myself for the menopause transition	49.5
I want to know more about symptoms	41.3
I am curious	35.6
I want to know more about treatment	35.5
I have symptoms	23.8
**Why do you NOT want information?** (*n* = 205)	%
I know what I need to know	42.0
I will take it as it comes	36.1
It is far ahead or I am too young	17.6
I am past menopause	13.2
I am not interested in the menopause transition	0.5
I am embarrassed	0.5
I fear what I will get to know	0
Other reasons	3.4

Participants could choose more than one answer.

In all, one-third did not want information (*n* = 205). The main reasons were that they already possessed enough information, would take menopause as it comes, thought they were too young, or they were already postmenopausal.

Participants with lower education reported a significantly lower need for information than those with higher education (*P*<0.05). Region of birth showed no influence on information needs.

Among the preferred information sources, the participants ranked the GP (67%), the internet (37%), and the gynaecologist (36%) as the most important (Table 4). When asked if they had already searched for information, almost half said they had, and less than half of these had found the information helpful. The main information sources used are listed in Table 4.

**Table 4.** Sources of information about menopause

	Where do you want information from? *n* = 366	Where have you previously searched for information? *n* = 292
	%	%
GP	66.9	8.6
Internet	37.2	68.9
Gyneacologist	35.5	5.8
Social media	14.8	1.7
Lectures	14.5	0
Friends	11.7	11
Books	9.8	3.1
Mother or family	9.3	1.7
Workplace	5.5	0
Other sources	4.6	8.3

Participants could choose more than one answer.

### The role of the GP

In total, 22% had enquired with their GP about menopause, and 10% reported that their GP had initiated the conversation. The information was deemed useful by 57% when self-enquired, whereas 86% found it helpful when the doctor initiated the discussion. When asked why their GP should address menopause, the main reason was *'Information to help myself in this period'* (Table 5). Among participants who wanted information, but whose GP had not addressed the topic, four out of five (81%) wanted their GP to do so. The sex of the GP did not influence this preference.

**Table 5.** Reasons for wanting the GP to address menopause, related to menopausal status among women who want information

Menopausal status	All, %*n* = 260	Pre-menopausal, %*n* = 166	Menopausal, %*n* = 55	Do not know, %*n* = 33
Information to help myself in this period	72.3	67.5	87.3	72.7
Preventive health information for good health in the future	61.9	58.4	67.3	66.7
Information from my GP who is a professional I trust	58.5	60.8	54.5	57.6
Information from the GP who will follow me further	40.4	36.1	45.5	54.5
Information from the GP who knows me	33.1	28.9	38.2	42.4

Participants could choose more than one answer

Participants were asked from what age they wanted their GP to address menopause; they answered from the median age of 45 years. Several specified that they wanted information before entering menopause and some as part of any consultation. When asked about situations during which the GP could address menopause, the predominant choice was when experiencing symptoms (84%), when coming to the GP for a cervical screening test (33%), or when discussing contraceptives (10%).

## Discussion

### Summary

This study has shown that nearly two-thirds of the participants desired menopause-related information, and four out of five preferred their GP to be involved. They wanted information before entering menopause, at a median age of 45 years. The GP’s sex did not influence the preference for information from the GP. Higher education increased the probability of wanting information. The main reason for wanting information was to help oneself in this period, and the preferred sources apart from the GP were the internet, friends, and the gynaecologist. One-third of women did not want information.

### Strengths and limitations

One strength of this study is the random female population addressed in this general practice setting. The women were in their GP’s waiting room for different reasons. Women aged between 30 and 60 years were asked, and most (86%) wanted to participate. We received fewer questionnaires than expected, as fewer students participated in distributing questionnaires than anticipated. The students reported difficulties in assessing women’s age (30–60 years). Thus, we had to exclude several questionnaires from the analyses. The women answered the questionnaire in their GP’s waiting room. This may have influenced their responses regarding the GP’s role in providing information. The research group created the questionnaire and the validation rested on the first author’s piloting and adjustments before the start of the study. The fact that only nine questionnaires out of 829 were classified as inconsistent suggests that the form was adequate in this context. The questionnaire was in Norwegian only and therefore excluded patients who could not read Norwegian. We included a group of women covering a wide ethnic background, as 100 women of the 625 women included were born outside Norway.

### Comparison with existing literature

The results support previous studies’ findings; women expressed need for information about menopause to be prepared and empowered and to improve future health.^5,8,21^

In our study, four out of five of those who wanted information would like their GP to address menopause, but only 10% reported that their doctors had initiated discussions about menopause with them. Studies have shown that women want and expect more information from their GP^5,6,21^ and that it is helpful to receive such information.^7^ Our results are transferable to other countries where the organisation of the healthcare system is similar, in which the GP is the first contact and a gatekeeper.

‘Time needed to treat’ is a concept to be considered when making guidelines^22^ and appears relevant in this context. We argue, however, that by discussing menopause and providing knowledge before symptoms, the GP can prevent worries and repeated visits later. This is supported by Herbert *et al*, who encourage clinicians to help women make informed health choices by being proactive in providing comprehensive information.^8^

Recent studies in the UK have shown that women and their GPs lack education about menopause.^5,21^ In Norway, Gjelsvik *et al* studied GPs’ attitudes and advice given to women concerning MHT in 2007.^16,23^ GPs were generally well-informed, but their opinions on whether menopause should be considered a natural phase or a medicalised issue influenced their approach to MHT.^16^ Providing information about menopause in general practice can be seen as a medicalisation, as it brings a natural stage of life into the healthcare system. However, it is important to note that medicalisation does not necessarily have to be negative.^24^ Receiving information from a GP can empower women before entering this life phase. Women described confusion about which symptoms are linked to menopause and what is normal in the middle of life, and they also expressed that they need care and information during this period.^25^

There is a commercial market for helping women during this period in life. It is argued that medicalisation happens when ‘*medicine meets the market*’.^26^ Receiving comprehensive, evidence-based information from their GP can assist women in navigating the overwhelming amount of advice on ageing and menopausal symptom relief prevalent in society. The GP is in a good position to help women, given their knowledge of previous medical history and background.

We found that the woman’s education level was related to her interest in information. This was also a result in a recent study in Iran,^14^ showing considerably increased interest with higher education.

There is also a need for other reliable information sources besides the GP. In our study, many participants had searched for information already, but less than half found it to be helpful. More than two-thirds had used the internet. Internet as a source of information is discussed in several studies.^5,14,21,27^ Women expressed confusion because the information is often inconsistent.^5^ There is a need for better quality websites addressing menopause.^5,27^ Several responders suggested public health campaigns directed towards women before entering menopause. This is supported by the findings of Munn *et al*, which suggest that public campaigns could help provide reliable information and stimulate discussion of the topic in society.^21^

### Implications for research and practice

This research has indicated that most women aged 30–60 years want information about menopause, they want to talk to their GP about it, and they want information before entering menopause. GPs need to make space for this conversation in daily practice. The information may include an overview of menopause, symptoms, up-to-date treatment options, and lifestyle recommendations for now and in the future. This might save women and their GPs time by empowering women in this period of life. It is important to note that one-third of the participants did not want information about menopause, which should be respected.

Further studies are needed to provide deeper insight into women’s information needs. Qualitative interviews with menopausal women experiencing distressing symptoms could help in exploring coping strategies, sources of information, and the perceived helpfulness of those sources.

## Data

The dataset relied on in this article is available from the corresponding author on reasonable request.
